# Genome-Wide Characterization of the GT4 Gene Family in Cotton

**DOI:** 10.3390/biology15191709

**Published:** 2026-09-25

**Authors:** Kai Zhang, Jiao Lin, Xin Wei, Zipiao Zheng, Liyun Yang, Chi Wang, Aerman Abulimiti, Yuanyuan Liu, Jiachen Wang, Yangdong Ye, Wenhua Ye, Shengjun Shen, Dawei Zhang, Haijiang Xu, Yang Jiao

**Affiliations:** 1 College of Agriculture, Tarim University/Key Laboratory of Genetic Improvement and Efficient Production of Characteristic Crops in Arid Regions of Southern Xinjiang, Aral 843300, China; z10757242012@163.com (K.Z.); linjiao@taru.edu.cn (J.L.); yuanyl4956@163.com (Y.L.); yeyangdong6246@163.com (Y.Y.); 18599703190@163.com (S.S.); 2 Xinjiang Key Laboratory of Cotton Genetic Improvement and Intelligent Production/Xinjiang Cotton Technology Innovation Center, Cotton Research Institute of Academy of Agricultural Sciences of Xinjiang Uyghur Autonomous Region (National Cotton Engineering Technology Research Center), Urumqi 830091, China; wx985688682@126.com (X.W.); zzpn001@126.com (Z.Z.); yangly@nwafu.edu.cn (L.Y.); 13055796278@163.com (C.W.); alove798@sina.com (A.A.); zbzdw012@126.com (D.Z.); 3 Agricultural College, Xinjiang Agricultural University, Urumqi 830052, China; wjc1402511951@163.com (J.W.); y2773239787@163.com (W.Y.)

**Keywords:** cotton, GT4, fuzz, abiotic stress, WGCNA, subcellular localization

## Abstract

The glycosyltransferase 4 (GT4) family plays essential roles in plant cell wall biosynthesis, glycolipid metabolism, and secondary metabolite glycosylation, yet its characterization in cotton remains scarce. Here, we performed a genome-wide identification of *GT4* genes in four cotton species using comprehensive bioinformatics approaches, and systematically analyzed their conserved motifs, gene structures, chromosomal distributions, phylogeny, and selection pressures. A total of 49 GT4 members were identified, including seven genes in *G. arboreum* (A2), nine genes in *G. raimondii* (D5), 16 genes in *G. hirsutum* (AD1), and 17 genes in *G. barbadense* (AD2). Phylogenetic analysis classified them into four subfamilies. Chromosomal mapping revealed uneven distribution and various duplication events. Promoter *cis*-element analysis indicated enrichment of light-, phytohormon-, and stress-responsive regulatory elements. Selection pressure analysis indicated predominant purifying selection among homologous pairs. Expression profiling across multiple tissues, fiber developmental stages, and four abiotic stresses (cold, drought, salt, and heat) identified *GhGT4_10* and *GhGT4_2* as stress-responsive candidate genes; *GhGT4_10* showed the most pronounced induction across the tested stresses, and both were drought-responsive by qRT-PCR. WGCNA under drought stress showed that the MEgreen module was most positively correlated with the 12 h post-treatment time point, and this module was significantly enriched in genes annotated to chloroplast components, photoresponse pathways, ribosomal machinery, and glyoxylate/dicarboxylate metabolism. Subcellular localization showed that *GhGT4_2* localizes to chloroplasts, consistent with its predicted localization; this observation supports a possible chloroplast-associated role but does not establish its function or involvement in a specific pathway. In fuzz development analysis, the MEturquoise module was highly correlated with fuzz phenotype, and a co-expression network centered on *GhGT4_15* identified 503 network-connected genes enriched in nucleocytoplasmic transport, phagosome, and spliceosome pathways. These enrichment results suggest a hypothesis that *GhGT4_15* may be associated with these pathways. qRT-PCR showed that *GhGT4_2* and *GhGT4_10* were drought-responsive, whereas *GhGT4_7* and *GhGT4_15* were associated with fuzz development and represent candidate regulators, including possible negative regulation, pending functional validation. Collectively, this study characterizes the structural and expression features of the cotton GT4 family and provides candidate genes for molecular breeding targeting abiotic stress resistance and fiber trait improvement; functional validation is needed to establish causal roles.

## Simple Summary

Cotton is an important cash crop worldwide, and its growth and yield are often threatened by abiotic stresses such as drought. The GT4 family proteins in plants are involved in cell wall synthesis, hormone signaling, and stress responses, but our understanding of GT4 genes in cotton has been very limited. In this study, we used bioinformatics approaches to identify a total of 49 *GT4* genes across four cotton species and systematically analyzed their structures, evolutionary relationships, and chromosomal distributions. Analysis of regulatory elements in the promoter regions revealed that several *GT4* genes may respond to light, phytohormones, and stress signals. Expression profiling and qRT-PCR showed that two genes, *GhGT4_2* and *GhGT4_10*, were significantly differentially expressed under drought treatment, identifying them as drought-responsive candidate genes for functional validation. Two other genes, *GhGT4_7* and *GhGT4_15*, were associated with cotton fuzz fiber development in expression and co-expression analyses, but whether they positively or negatively regulate fuzz development remains to be determined. We also observed that the *GhGT4_2* protein localized to chloroplasts, consistent with its predicted localization. This study provides candidate gene resources and a theoretical basis for future molecular breeding aimed at improving stress tolerance and fiber quality in cotton.

## 1. Introduction

Glycosylation is widely present in both prokaryotes and eukaryotes and represents a crucial post-translational modification [1,2]. *Glycosyltransferases* (GTs), which mediate this process, can transfer activated sugar donors to various small-molecule acceptors, thereby affecting the stability and solubility of the acceptor molecules; moreover, due to substrate diversity and the existence of multi-site binding, this modification may also confer rich structural diversity to the products [3].

*Glycosyltransferases* (abbreviated as GTs, enzyme commission number EC 2.4.x.y) are ubiquitously distributed in diverse organisms. As key enzymes in carbohydrate metabolism, they catalyze the transfer of glycosyl groups from activated donor molecules to various acceptors, including carbohydrates, lipids, and proteins, thereby forming glycosidic linkages [2]. Following glycosylation, the solubility, chemical stability, toxicity, and bioactivity of the acceptors are typically altered significantly [4]. GTs are extremely common in the plant kingdom, spanning a broad range of taxa from lower plants such as Chlamydomonas reinhardtii and Physcomitrella patens to higher plants like *Arabidopsis thaliana* and maize, and this widespread occurrence underscores their important biological significance [5]. Based on amino acid sequence similarity, GTs have been classified into more than 100 families [5], with each family exhibiting remarkable diversity in three-dimensional structure and catalytic function [6,7]. For instance, proteins of the GT1 family all contain a 44-residue PSPG motif (the plant secondary product *glycosyltransferase* signature sequence), and some members have been shown to participate in the biosynthesis of plant natural products [8], while others are associated with fertility processes in maize [9]. Proteins of the GT8 family possess a *Glyco_transf_8* domain of approximately 256 amino acids, and studies have indicated that some members of this family function in plant cell wall biosynthesis [7]. Given that the GT families encompass a large number of subfamilies and numerous genes, comprehensively annotating the biological function of each member in diverse target plants remains a formidable task in plant science. To address this challenge, a bioinformatics strategy integrating whole-genome identification, phylogenetic analysis, and expression profiling has emerged in recent years and has been widely applied for the systematic dissection of specific gene family functions. Notably, a systematic genome-wide characterization of the GT4 gene family across diploid and allotetraploid cotton species remains lacking.

Cotton, as the world’s most important natural fiber source, is not only the core raw material for the textile industry but also occupies a key position in the current global economic system [10,11]. Its superior fiber quality traits have prompted in-depth research into the molecular regulatory mechanisms underlying fiber development and genetic improvement strategies, with the aim of effectively enhancing cotton fiber yield and quality through modern biotechnological approaches. However, cotton plants are often subject to various adverse environmental factors throughout their growth cycle, including abiotic stresses such as drought, salinity, low temperature, and high temperature, as well as biotic stresses like pests and diseases [12,13,14]. These unfavorable conditions severely inhibit normal plant development, ultimately leading to yield losses and fiber quality deterioration. The rise in genetic engineering technology has provided effective avenues to address these challenges, and the ongoing progress in cotton genome sequencing and assembly has laid a solid foundation for systematic studies of various gene families in *Gossypium* species [15,16,17].

Despite the established roles of GT4 family proteins in plant cell wall biosynthesis, hormone signaling, and stress responses, knowledge of this family in cotton remains limited. In particular, GT4 genes have not been systematically characterized across cotton species, and their functions in abiotic stress tolerance and fuzz development are largely unknown. Our preliminary analysis of publicly available transcriptome data [17] suggested that some GT4 family members are differentially expressed under drought treatment and that several others may be associated with fiber development. These observations prompted us to perform a comprehensive investigation of the GT4 family in cotton. To this end, we selected two diploid cotton species, *G. arboreum* and *G. raimondii*, and two tetraploid species, *G. hirsutum* and *G. barbadense*, and aimed to: (1) identify and classify all GT4 family members in these four species; (2) characterize their phylogenetic relationships, gene structures, conserved motifs, chromosomal distributions, and selection pressures; (3) predict promoter *cis*-elements and analyze tissue-specific and stress-responsive expression patterns; (4) construct co-expression networks associated with drought stress and fuzz development; and (5) validate candidate genes by qRT-PCR and subcellular localization. This study provides a systematic catalog of the cotton GT4 gene family and candidate gene resources for future functional validation and molecular breeding aimed at improving abiotic stress tolerance and fiber quality in cotton.

## 2. Materials and Methods

### 2.1. Identification of Cotton GT4 Gene Family Members

Reference genomes and genome annotation GFF files for *G. arboreum* (ICR), *G. barbadense* (ZJU), *G. raimondii* (JGI), and *G. hirsutum* (ZJU) were downloaded from the CottonMD database (https://yanglab.hzau.edu.cn/CottonMD, accessed on 8 February 2026) [18]. Using the *Arabidopsis* GT4 protein sequences, we performed Local BLASTP (https://blast.ncbi.nlm.nih.gov/Blast.cgi?PAGE=Proteins, accessed on 8 February 2026) searches to identify GT4 genes in the four cotton species. The identified GT4 genes were validated using the HMM (Hidden Markov Model) profile obtained from the Pfam (PF13439) database (https://www.ebi.ac.uk/interpro/entry/pfam/PF13439/proteome/uniprot/, accessed on 8 February 2026) [19]. Domain information of the identified GT4 proteins was further confirmed through NCBI Batch-CDD searches. To investigate the physicochemical properties of the GT4 genes, we used the TBtools-II software (version 2.5) for analysis [20].

### 2.2. Chromosomal Locations and Gene Duplication Analysis of GT4 Genes

To examine the chromosomal positions of the GT4 genes in the four *Gossypium* species, the physical chromosome locations of all GT4 genes were visualized using TBtools-II software (version 2.5) [20], and MCScanX (version 1.0.0)was used to analyze genomic synteny blocks and gene duplication events [21].

### 2.3. Phylogenetic Reconstruction of GT4 Family Proteins

To elucidate the evolutionary relationships among GT4 homologs across four cotton species (*G. arboreum*, *G. hirsutum*, *G. raimondii*, and *G. barbadense*), a multiple sequence alignment of the 49 identified GT4 genes was carried out using ClustalW within the MEGA7 platform (https://www.megasoftware.net/, accessed on 8 February 2026) [22]. Based on this alignment, a maximum-likelihood (ML) phylogenetic tree was inferred, with nodal support assessed via 1000 bootstrap replicates.

### 2.4. Structural and Motif Characterization of GT4 Family Genes

For visualization of gene architecture and conserved protein motifs, the MEME-derived results, the phylogenetic tree in NWK format, and the genome annotation file (GFF3) of *G. hirsutum* were integrated and displayed using TBtools-II software (version 2.5) [20].

### 2.5. Expression Dynamics and Promoter cis-Element Composition of the GT4 Gene Family

RNA-seq data of *G. hirsutum* cultivar TM-1 were downloaded from NCBI (https://www.ncbi.nlm.nih.gov/, accessed on 8 February 2026). These data were used to analyze the expression of *GhGT4* genes in eight different tissues as well as at various fiber and ovule developmental stages. RNA-seq data of *G. hirsutum* TM-1 under cold, heat, salt, and PEG stresses were also downloaded [17].

Total RNA was extracted from ovules and fibers of XNZ118 and XNZ118LM at 0, 1, 3, 4, and 5 days post anthesis (DPA) using the RNAprep Pure Plant Kit (Polysaccharides & Polyphenolics-rich) from Tiangen Biotech (Beijing, China) Co., Ltd., with three biological replicates per sample. Ovule samples were collected at 0, 1, and 3 DPA, whereas a mixture of fibers and ovules was collected at 4 DPA and 5 DPA. RNA samples that passed quality assessment were then sent to Biomarker Technologies (Beijing, China) Co., Ltd. for cDNA library construction. For this transcriptomic dataset, a total of 30 cDNA libraries were successfully constructed. The cDNA libraries were sequenced on the Illumina (San Diego, CA, USA) high-throughput sequencing platform to obtain raw data. After removing low-quality reads and adapter sequences, the resulting clean reads were mapped to the *Gossypium hirsutum* TM-1 (v 2.1) reference genome [17]. Pearson correlation coefficients were used to evaluate the biological reproducibility and correlation of the samples, indicating that the biological replicates were reliable (Figure S1). Therefore, the sample quality of this transcriptomic dataset was reliable and could be used for subsequent analyses (Supplementary Table S1).

To preliminarily explore the functions of gene expression regulation, we extracted the 2.0 kb sequences upstream of the start codons of the GT4 family genes in *G. hirsutum* as promoter sequences for *cis*-element analysis. *Cis*-elements in the GT4 gene promoter regions were further analyzed using PlantCARE website (https://bio.tools/plantcare, accessed on 8 February 2026), and the obtained *cis*-acting element information was visualized with TBtools-II software (version 2.5) [20].

### 2.6. Synteny and Selection Pressure Calculation of GT4 Family Genes

To explore the evolutionary relationships and selection pressures of the GT4 family genes in the four cotton species, genes with syntenic relationships were identified across the whole genomes by BLASTing all cotton protein sequences and comparing them using MCScanX [21]. Finally, TBtools-II software (version 2.5) was used for visualization [20], and the same software was used to calculate the non-synonymous (Ka) and synonymous (Ks) substitution rates of duplicated genes [20].

### 2.7. WGCNA of Fuzz Materials and Drought Stress Materials

Using the WGCNA package (version 3.3) [23] in R (version 4.5.3) to construct co-expression networks, we used two independent transcriptomic datasets. The first (fuzz dataset) comprised 30 fiber samples from unpublished fuzz(XNZ118) and fuzzless(XNZ118 FLM) *G. hirsutum* lines. XNZ118 FLM is a natural fuzzless mutant of XNZ118. These 30 samples included three independent biological replicates at (0, 1, 3, 4, 5) DPA. Genes with FPKM > 5 in samples were retained. Outlier samples were assessed by PCA and hierarchical clustering. A *β* of 7 was selected because it achieved a scale-free topology fit (R^2^ > 0.9) and reasonable mean connectivity. The adjacency matrix was converted to a topological overlap matrix, followed by average-linkage hierarchical clustering, dynamic tree cutting, and module merging. A minimum module size of 30 genes was used.

The second independent WGCNA analysis was based on the publicly available TM-1 drought-stress transcriptome dataset [17]. All genes with FPKM > 3 were selected. In this case, outlier samples were assessed by PCA and hierarchical clustering. *β* of 9 was chosen because it achieved a scale-free topology fit (R^2^ > 0.9) and had a reasonable mean connectivity. The adjacency matrix was converted to a topological overlap matrix, followed by average-linkage hierarchical clustering, dynamic tree cutting, and module merging. A minimum module size of 30 genes was used.

### 2.8. GO and KEGG Enrichment Analysis and Co-Expression Network Construction

For functional annotation, genes assigned to the modules of interest were queried against the GO and KEGG pathway database using TBtools-II software (version 2.5) [20]. Enrichment significance was determined with stringent thresholds of *p* < 0.01 and Q < 0.05.

### 2.9. qRT-PCR Analysis

We examined the expression levels of four *GhGT4* genes (*GhGT4_2*, *GhGT4_7*, *GhGT4_10*, and *GhGT4_15*) under PEG-simulated drought stress in two upland cotton lines, KK1543 (drought-tolerant) and XLZ26 (drought-sensitive), and during fuzz development in GZNn and its fuzzless mutant GZNn FLM. GZNn FLM is a natural fuzzless mutant of GZNn, GZNn FLM is a natural fuzzless mutant of GZNn, and XNZ118 FLM is phenotypically similar to GZNn FLM, with both exhibiting a fuzzless phenotype. Seeds of KK1543 and XLZ26 were germinated at 28 °C under a 16 h light/8 h dark photoperiod and then transferred to a normal hydroponic culture system. Hoagland nutrient solution was added every two days. At the two-leaf stage, drought treatment was applied using 15% PEG6000. Leaf samples were collected at 0, 6, 12, and 24 h after treatment. For fuzz development, ovule and fiber samples were collected from GZNn and GZNn FLM at four stages (0, 1, 3, and 5 DPA). Three independent biological replicates were performed for each sample, and each biological replicate consisted of three technical qPCR replicates.

Total RNA was extracted from leaves or ovules/fibers using TRIzol (Eric Biotechnology), treated with DNase I, and reverse-transcribed from 1 µg of total RNA using the PrimeScript RT kit (Vazyme, Nanjing, China). Real-time PCR was performed on a 7500 Real-Time PCR System. Relative expression levels were calculated using the 2^−ΔΔCt^ method [24,25], where ΔCt = Ct(target gene) − Ct(GhUBQ7) and ΔΔCt = ΔCt(treated sample) − ΔCt(control sample). For each biological replicate, the Ct values of the three technical replicates were averaged before calculating ΔCt. The averaged ΔCt values from the three biological replicates were used as the statistical units. Statistical significance was determined by two-way ANOVA followed by Tukey’s HSD multiple comparison test. Asterisks indicate significant differences (* *p* < 0.05, ** *p* < 0.01, *** *p* < 0.001, **** *p* < 0.0001), and ns indicates no significant difference. A complete list of primers is provided in Supplementary Table S2.

### 2.10. Subcellular Localization of GhGT4_2 Protein

To determine the subcellular localization of *GhGT4_2* protein, the coding sequence of *GhGT4_2* was fused in-frame to the C-terminus of GFP under the control of the CaMV 35S promoter in the pCAMBIA1300-GFP vector (*GhGT4_2*-GFP). The construct was transformed into *Agrobacterium tumefaciens* strain LBA4404 and cultured at 30 °C for two days. The bacterial suspension was then infiltrated into *Nicotiana benthamiana* leaves. As a control, the free GFP construct (pCAMBIA1300-GFP without the *GhGT4_2* insert) was processed in parallel. After infiltration, the plants were kept under subdued light for 48 h. Tissue samples from the infiltrated areas were prepared on slides and visualized using a confocal laser-scanning microscope. GFP fluorescence was excited at 488 nm and detected at 500–530 nm. Chlorophyll autofluorescence was excited at 488 nm and detected at 650–700 nm. All images were captured with identical settings.

## 3. Results

### 3.1. Genomic Identification of GT4 Genes

Candidate sequences were obtained by searching the target files using the local BLASTP program. After validating the conserved domains with Pfam and CDD, sequences lacking the complete GT4 domain were discarded. Ultimately, 49 GT4 genes were identified in *G. arboreum*, *G. raimondii*, *G. barbadense*, and *G. hirsutum* (Supplementary Table S3), including seven genes in *G. arboreum* (A2), nine genes in *G. raimondii* (D5), 16 genes in *G. hirsutum* (AD1), and 17 genes in *G. barbadense* (AD2). Notably, the D-subgenome species *G. raimondii* (D5) had two more genes than the A-subgenome species *G. arboreum* (A2). The sum of gene numbers in the two diploid cotton species (*G. arboreum* and *G. raimondii*) was less than that in *G. barbadense* and exactly equal to that in *G. hirsutum*. Subsequently, based on their chromosomal positions, these genes were renamed *GaGT4_1-GaGT4_7*, *GhGT4_1-GhGT4_16*, *GrGT4_1-GrGT4_9*, and *GbGT4_1-GbGT4_17* (Supplementary Table S3).

Next, we assessed the amino-acid properties of GT4 homologs across the four *Gossypium* species. Lengths extended from 392 to 1153 aa, averaging 481 aa; molecular masses fell in the range of 43.51–131.42 kDa, with a mean of 54.08 kDa; and pI values ranged from 6.06 to 9.59, the average being 8.23. Full details appear in Supplementary Table S3.

### 3.2. Phylogenetic Analysis of GT4 Genes

To analyze the evolutionary relationships among GT4 family members, a phylogenetic tree was constructed using MEGA7 based on the 49 GT4 protein sequences from the four cotton species (Figure 1). Ultimately, the 49 GT4 family members were divided into four distinct subfamilies, designated Class1-Class4. Among them, Class3 contained the largest number of members, with 14, followed by Class1 and Class4, each with 13, and Class2 had the fewest, with nine. Interestingly, the numbers of *G. hirsutum* and *G. barbadense* genes in Class1 and Class3 were identical; in Class4, *G. hirsutum* had more genes than *G. barbadense*, whereas in Class2, *G. barbadense* had more genes than *G. hirsutum*, and no *G. raimondii* genes were found in Class2. It is noteworthy that the GT4 sequences from the two diploid species consistently clustered with the corresponding sequences from the tetraploid species. This clustering pattern is consistent with the hypothesis that tetraploid cotton species originated from hybridization and polyploidization between A- and D-genome diploid lineages represented by *G. arboreum* and *G. raimondii*.

### 3.3. Chromosomal Location of GT4 in Four Cotton Species

To further investigate the chromosomal distribution and gene duplication events of the GT4 genes in the four cotton species, we mapped their physical positions on the chromosomes. The 49 GT4 genes were distributed across the chromosomes of these four species, as shown in Figure 2. In *G. hirsutum*, 16 genes were located on 12 chromosomes, namely A01, A03, A04, A09, A11, A12, D01, D03, D04, D05, D11, and D12. Each chromosome carried one or two GT4 genes; chromosomes A01, A04, D01, and D03 each contained two genes, with a tandem duplication detected on D03, while the remaining chromosomes each harbored a single gene. In *G. hirsutum*, the A subgenome contained eight genes, and coincidentally, the D subgenome also contained eight genes. The distribution pattern in *G. barbadense* was similar to that in *G. hirsutum*, with genes located on the corresponding chromosome numbers, except that chromosome A12 carried two GT4 genes in *G. barbadense* whereas only one in *G. hirsutum*. In *G. arboreum*, seven GT4 genes were distributed across four chromosomes, Chr01, Chr04, Chr05, and Chr12; Chr01 contained the highest number with three genes, and a tandem duplication was found on Chr01, while each of the other chromosomes had one gene. Similarly, in *G. raimondii*, nine GT4 genes were distributed across seven chromosomes, Chr01, Chr02, Chr04, Chr05, Chr10, Chr11, and Chr12, with Chr01 and Chr02 each containing two genes, and a tandem duplication was present on Chr02.

### 3.4. Gene Structure, Protein Motif Analysis, and cis-Acting Element Analysis

To further explore the potential structural evolutionary links among GT4 family members, we constructed a phylogenetic tree for the 16 GT4 genes of *G. hirsutum* based on the maximum likelihood method, and concurrently performed motif association analysis and gene structure dissection, as shown in Figure 3. By integrating the protein sequences and annotation files of these genes, we obtained phylogenetic relationships and gene structure information. The conserved motifs of the GT4 proteins were identified using MEME and TBtools-II software (version 2.5). Among the 16 members in *G. hirsutum*, a total of 10 conserved motifs were recognized. Among them, *GhGT4_4*, *GhGT4_5*, *GhGT4_13*, *GhGT4_14*, *GhGT4_8*, *GhGT4_16*, *GhGT4_2*, *GhGT4_10*, *GhGT4_3*, and *GhGT4_12* contained the largest number of motifs, each harboring eight motifs. *GhGT4_6* contained the fewest motifs, with only three. In addition, we analyzed the intron-exon structural features. As shown in Figure 3, genes within the same group shared similar intron-exon arrangements. Among them, *GhGT4_7* contained the highest number of exons, with 17 exons and 16 introns. *GhGT4_6* contained 15 exons and 14 introns, and possessed the longest intron.

To gain a deeper understanding of the regulatory mechanisms of GT4 genes, we predicted *cis*-acting elements in the promoter regions of the 16 GT4 genes in *G. hirsutum* using the PlantCARE database, focusing on the 2000 base pairs upstream of the start sites. In the present study, at the level of hormone regulatory networks, we identified *cis*-acting elements responsive to methyl jasmonate, salicylic acid, gibberellin, and abscisic acid, and notably also found associated motifs involved in ABA signal transduction. In terms of abiotic and biotic stress responses, we identified modules responsive to low temperature, MYB recognition sites inducible by drought, regulatory sequences associated with defense and stress responses, and core *cis*-elements essential for anaerobic conditions. In the dimension of light signal transduction, in addition to canonical light-responsive *cis*-regulatory units, we also detected light-responsive specific sites bound by MYB-type transcription factors. Furthermore, we resolved regulatory motifs associated with endosperm-specific expression, meristem activity maintenance, and MYBHv1 binding function. The systematic identification of these diverse *cis*-elements provides a crucial molecular basis for functional elucidation of this gene family in complex biological processes such as developmental regulation, environmental adaptation, and metabolic integration [26].

### 3.5. Exploration of Gene Duplication Events and Syntenic Associations

The evolutionary pathways of gene families mainly include whole-genome duplication (WGD) or segmental duplication, dispersed duplication, tandem duplication, and proximal duplication [27]. To investigate the evolutionary patterns and effects of polyploidization, we identified the duplication types of GT4 genes in the four cotton species (Supplementary Table S4). In the diploid species, *G. arboreum* had two genes derived from WGD or segmental duplication, three genes from dispersed duplication, and two genes from tandem duplication; *G. raimondii* had three genes from WGD or segmental duplication, three genes from dispersed duplication, two genes from tandem duplication, and one gene from proximal duplication. In the tetraploid species, *G. hirsutum* had 14 genes derived from WGD or segmental duplication, 1 gene from tandem duplication, and 1 gene from proximal duplication. In *G. barbadense*, two genes were from tandem duplication, and all the remaining genes were from WGD or segmental duplication. Thus, in the tetraploid cotton species, the majority of genes evolved via WGD or segmental duplication, whereas in the two diploid species, they were mainly distributed among WGD or segmental, dispersed, and tandem duplications.

We performed multiple synteny analyses of GT4 genes across *G. barbadense* (AD2), *G. hirsutum* (AD1), *G. arboreum* (A2), and *G. raimondii* (D5) (Figure 4E). We found 12 orthologous gene pairs between *G. hirsutum* and *G. arboreum*, 10 orthologous pairs between *G. hirsutum* and *G. raimondii*, and 13 orthologous pairs between *G. hirsutum* and *G. barbadense*.

In addition, we analyzed intra-genomic synteny within tetraploid *G. hirsutum* (Figure 4C) and identified 12 orthologous/paralogous pairs. Similarly, we identified one orthologous/paralogous pair within the *G. arboreum* genome (Figure 4A), two pairs within the D-genome *G. raimondii* (Figure 4B), and 14 pairs within *G. barbadense* (Figure 4D).

### 3.6. Calculation of Selection Pressure

To understand how GT4 genes diverged following polyploid events in cotton, we calculated Ka/Ks ratios for 100 homologous pairs from the four species. Across *G. arboreum*, *G. raimondii*, *G. hirsutum*, and *G. barbadense*, every ratio was below 1 (Supplementary Table S5). Such universal sub-unity values are consistent with these genes having been predominantly shaped by purifying selection; however, this analysis does not exclude the possibility of positive selection at specific codons or branches (Figure 4F).

Furthermore, we also calculated the Ka/Ks ratios between the two diploid and the two tetraploid cotton species (Figure 4F). The results showed that all homologous gene pairs had Ka/Ks ratios less than 1, suggesting that the GT4 genes analyzed were predominantly subject to purifying selection. Because these ratios are gene-wide averages, they should not be interpreted as evidence for equal selection strength across all pairs or as excluding lineage-specific positive selection.

### 3.7. Expression Profiles of GT4 Genes Within G. hirsutum

Gene expression patterns are closely associated with their biological functions. To analyze the expression differences of GT4 family members across various tissues, we examined transcriptome data from *G. hirsutum* tissues [17]. The results (Figure 5A) showed that *GhGT4_10* exhibited the highest expression in the calycle, while *GhGT4_2* also showed relatively high expression in the calycle and torus. *GhGT4_9* had the highest expression in petals, whereas some members such as *GhGT4_3*, *GhGT4_4*, and *GhGT4_11* were almost undetectable in multiple tissues. Notably, *GhGT4_5* and *GhGT4_7* displayed prominent expression in roots and pistils. In addition, *GhGT4_11* showed no expression in any tissues. These members exhibited diverse spatial expression patterns, suggesting that they may assume differentiated functions in different cotton organs.

Based on the expression profiles of this gene family at different developmental stages of cotton ovules and fibers (Figure 5B), the transcripts showed clear temporal and spatial dynamics. Overall, most transcripts exhibited higher expression levels during early ovule development (from −3 to 1 DPA), which gradually decreased as development progressed. For instance, *GhGT4_2*, *GhGT4_10*, and *GhGT4_15* peaked at −3 DPA (Days Post-Anthesis) or 1 DPA and then dropped markedly after 10 DPA, but rebounded in fibers, especially at 20 or 25 DPA, suggesting their possible involvement in fiber elongation or secondary wall thickening. *GhGT4_7* maintained relatively high and stable expression throughout ovule development and also remained at high levels in 5 DPA fibers, indicating a potential continuous function across ovule and fiber development. It is noteworthy that *GhGT4_11* showed no expression in all samples, while *GhGT4_3* and *GhGT4_4* were expressed at extremely low levels only at a few time points, possibly representing tissue-specific or inactive transcripts. Overall, the expression divergence of this family members during ovule and fiber development implies that they may coordinately regulate different biological processes such as fiber initiation, elongation, and maturation.

We then used transcriptome data from fuzz materials (XNZ118) and fuzzless materials (XNZ118 FLM) development in *G. hirsutum* (Figure 5C) and found that at 3 DPA and 4 DPA, *GhGT4_5* expression increased in the fuzzless material but decreased in the fuzz variety, suggesting a possible negative regulatory role in fuzz formation. Conversely, *GhGT4_9* expression increased in the fuzz variety but decreased in the fuzzless material, indicating a potential positive promotion of fuzz development. Moreover, *GhGT4_15* displayed contrasting expression trends between fuzz and fuzz materials at 1 DPA, 3 DPA, and 4 DPA, which might provide a basis for fuzz differentiation.

Next, based on the publicly available transcriptome data of the upland cotton material TM-1 under abiotic stresses (low temperature, drought, salt stress, and heat stress at different time points) [17], we found that (Figure 5D), *GhGT4_10* showed the most pronounced induction among all transcripts analyzed under the tested conditions. Its expression peaked at 6 h under cold stress, at 12 h under drought and salt stress, and at 24 h under heat stress. These observations support *GhGT4_10* as a stress-responsive candidate gene with possible broad-spectrum relevance; however, they do not demonstrate that it functions as a broad-spectrum stress regulator. *GhGT4_2* was also strongly induced under cold stress at 6 h and under salt stress at 6 h and 24 h, but was suppressed under heat treatment, showing an expression pattern consistent with stress-type-specific responsiveness. *GhGT4_1* was continuously upregulated under cold treatment and rose sharply at 1 h of heat stress, whereas its responses to drought and salt were relatively moderate. *GhGT4_5* and *GhGT4_6* showed mild induction under multiple stresses, especially at later time points under drought and heat. Notably, *GhGT4_3*, *GhGT4_11*, *GhGT4_12*, and *GhGT4_14* were almost undetectable or expressed at extremely low levels under all tested stress conditions, showing no clear evidence of stress responsiveness under these conditions; this does not exclude possible roles in other tissues, developmental stages, or stress regimes. Overall, based on expression profiling, *GhGT4_10* and *GhGT4_2* represent the most stress-responsive candidates in this family, with expression dynamics dependent on stress type and duration. Their expression patterns were associated with cold, drought, salt, and heat responses, and they represent candidates for functional validation to determine whether they contribute to cotton adaptation to adverse environments.

Based on the expression profiles of this gene family in the Verticillium wilt-susceptible material JM1 and the resistant material SDLK from 0 to 48 h post-inoculation (Figure 5E), *GhGT4_5* exhibited the most prominent variety-specific induction. In the susceptible material, *GhGT4_5* expression increased sharply from 0 h to 24 h, reaching its highest level at 24 h, and remained relatively high at 48 h; whereas in the resistant material, its expression also increased but to a much lesser extent and declined after 24 h, suggesting that *GhGT4_5* may positively respond to pathogen infection signals and participate in defense or susceptibility-related regulatory pathways during disease development. *GhGT4_13* also showed significant sustained upregulation in the susceptible material but declined after 12 h in SDLK, further supporting its close association with the response of susceptible materials to *Verticillium dahliae*. Overall, the specific and substantial upregulation of *GhGT4_5* and *GhGT4_13* in the susceptible material JM1 indicates that they may be key response factors during the early (12–24 h) and sustained (48 h) stages of Verticillium wilt infection, providing important clues for screening candidate genes related to Verticillium wilt resistance in cotton.

### 3.8. WGCNA of GhGT4 Members in G. hirsutum

Although the evolutionary relationships, sequence structures, synteny analyses, selection pressure, and expression patterns of the GT4 gene family have been extensively studied, and transcriptome data have been used to examine *GhGT4s* across various tissues, different fiber developmental stages, and four abiotic stresses, their potential roles in drought tolerance and fuzz development remain unclear. We therefore used publicly available transcriptome data for drought stress [17] and unpublished transcriptome data for fuzz development (Transcriptome data of ovules and fiber samples from five developmental stages of the fuzzless mutant XNZ118 FLM and XNZ118). Genes with FPKM values greater than 3 (for drought transcriptome) and greater than 5 (for fuzz transcriptome) were selected, yielding 20,609 and 20,601 genes, respectively, for WGCNA.

The dynamic tree-cut procedure, combined with weight-guided merging of similar expression modules, yielded 16 modules for *G. hirsutum* line TM-1 (Figure 6A,B). Among these, core modules were distinguished by the thresholds |r| > 0.8 and *p* < 0.0001 (Figure 6B). Among them, the MEgreen module (r = 0.83, *p* < 0.000016) showed a significant positive correlation with the 12 h time point after drought stress. Notably, among the 16 members of the GT4 family in *G. hirsutum*, the genes *GhGT4_2* and *GhGT4_10* belonged to the MEgreen module. We first performed GO enrichment analysis for this module (Figure 6C), and found that in Biological Process, the main enrichments were in plastid organization (GO:0009657), photosynthesis, light reaction (GO:0019684), and photosynthesis (GO:0015979); in Cellular Component, the main enrichments were in chloroplast part (GO:0044434), plastid part (GO:0044435), and chloroplast (GO:0009507); in Molecular Function, the main enrichments were in structural constituent of ribosome (GO:0003735), structural molecule activity (GO:0005198), and aminoacyl-tRNA ligase activity (GO:0004812). Subsequently, KEGG enrichment analysis (Figure 6D) revealed that this module was mainly enriched in pathways such as aminoacyl-tRNA biosynthesis, ribosome, and glyoxylate and dicarboxylate metabolism. Based on these enrichment results, the presence of *GhGT4_2* and *GhGT4_10* in this drought-associated module showed a co-expression pattern consistent with a possible association with drought response; however, this pattern does not establish regulatory direction or pathway-specific function. Their co-expression with genes enriched in energy maintenance, protein synthesis, and metabolic remodeling—including chloroplast stability, photosynthetic light reactions, the ribosomal translation machinery, and glyoxylate metabolism—is consistent with possible involvement in drought-related processes, but these proposed roles represent hypotheses that require functional validation.

In the fuzz (XNZ118) and fuzzless (XNZ118 FLM) materials, we selected genes with FPKM > 3 for WGCNA analysis, a total of 11 modules were obtained (Figure 7A). Following the criteria of |r| > 0.7 and *p* < 0.0001, we screened for candidate modules associated with fuzz development among the GT4 family members (Figure 7B). Among the 16 GT4 family members in *G. hirsutum*, three genes, namely *GhGT4_7*, *GhGT4_9*, and *GhGT4_15*, belonged to the MEturquoise module. We found that the MEturquoise module (r = 0.71, *p* < 0.000014) showed a significant correlation with fuzz development traits. In our previous analyses, GO and KEGG enrichment analyses had already been performed for this module. Based on the above fuzz transcriptome data, we therefore further analyzed the co-expression network of *GhGT4_15* using a weight threshold of 0.3 (Figure 7C). A total of 503 unique network-connected genes (nodes) were retained for construction of the co-expression network (Supplementary Table S6). We then performed KEGG analysis on the genes co-expressed with *GhGT4_15* (Figure 7D). The results showed that these network-connected genes were mainly enriched in pathways such as nucleocytoplasmic transport, phagosome, and spliceosome. These enrichment results generate hypotheses about possible pathways associated with *GhGT4_15* but do not demonstrate that *GhGT4_15* regulates these pathways. Whether *GhGT4_15* acts through this pathway remains to be functionally tested.

### 3.9. Subcellular Localization Analysis of GhGT4_2

To determine the subcellular localization of *GhGT4_2* protein, the coding sequence of *GhGT4_2* was fused in-frame to the C-terminus of GFP and free GFP as a control in tobacco epidermal cells. The results (Figure 8) showed that *GhGT4_2* protein was predominantly localized in chloroplasts.

### 3.10. Expression Analysis of GhGT4s in Different Cotton Varieties

To investigate the expression levels of *GhGT4s* under drought stress conditions and during fuzz development, we integrated the results of previous expression analyses and *cis*-acting element studies, and proposed that *GhGT4_2*, *GhGT4_7*, *GhGT4_10*, and *GhGT4_15* may play roles in drought stress responses and in the regulation of fuzz development. To further elucidate these functions, we selected several *G. hirsutum* varieties for expression pattern analysis and monitored the expression dynamics of these genes across different cotton materials and time points. We used upland cotton lines Xinluzao 26 and KK1543 for qPCR-based expression validation under PEG stress, and we used upland cotton line GZNn and its fuzzless mutant GZNn FLM for qPCR-based expression validation during fuzz development stages.

First, under PEG-induced drought stress, as shown in Figure 9A, the transcript levels of *GhGT4_2* and *GhGT4_10* indicated their potential involvement in the drought response of *G. hirsutum*. The expression of *GhGT4_2* remained generally low (<2.5) in both Xinluzao 26 and KK1543, with a significant difference between the two materials only at the later stage of drought stress, specifically at 24 h (*p* < 0.05), whereas no significant differences were detected at 0, 6, and 12 h. In contrast, *GhGT4_10* was significantly upregulated at 6 and 12 h in Xinluzao 26, peaked at 12 h (relative expression level ~5.5), and then declined rapidly at 24 h. Although this gene was also upregulated in KK1543, the magnitude was significantly lower than that in Xinluzao 26. At 6 and 12 h, the differences between the two materials were highly significant (*** *p* < 0.0001), whereas no significant differences were observed at 0 and 24 h.

Subsequently, we examined the expression of *GhGT4_7* and *GhGT4_15* during fuzz development. As shown in Figure 9B, *GhGT4_7* exhibited a pulse-like expression trend of first increasing and then decreasing in both materials. At 1 DPA, the expression level in GZNn was significantly higher than that in GZNn FLM (* *p* < 0.0001); at 3 DPA, the expression level in GZNn FLM was significantly higher than that in GZNn (*p* < 0.01); and by 5 DPA, the expression levels in both groups had declined with no significant difference (ns). *GhGT4_15* displayed a single-peak curve that first increased and then decreased in both materials, peaking at 3 DPA. Notably, the expression level in GZNn FLM was significantly higher than that in GZNn at 1 DPA (*p* < 0.001), 3 DPA (*p* < 0.01), and 5 DPA (** *p* < 0.01), indicating that the transcriptional activity of this gene was continuously enhanced in the GZNn FLM background during early fiber development (1–5 DPA). It should be noted that the absence of the 4 DPA time point from the qRT-PCR analysis was not an omission but a targeted validation design. The 0, 1, and 3 DPA time points cover the ovule/fiber initiation stages, and 5 DPA covers the early fiber elongation stage; together, these time points are sufficient to validate the expression trends of the candidate genes.

These results highlight the potential roles of *GhGT4_2* and *GhGT4_10* in drought stress responses, and suggest that *GhGT4_7* and *GhGT4_15* may be associated with fuzz development in *G. hirsutum*.

## 4. Discussion

Cotton is not only an important economic source of natural fiber but also an ideal material for polyploidy genetics research [28]. During field growth and maturation, it frequently encounters abiotic stresses such as drought, high salinity, and extreme temperatures (cold and heat), which significantly inhibit normal growth rhythms and consequently lead to yield reduction and fiber quality deterioration. To overcome this challenge, researchers have focused on improving crop stress tolerance and thereby enhancing cotton fiber quality. With the emergence of whole-genome sequencing and chromosome-level assembly in cotton, systematic exploration of gene families in *Gossypium* has become well-prepared. To date, several families have been studied, including *GbTubulin* [29], *GbTPX2* [30], *GhGT92* [31], *GhFWL* [32], *GhABI3* [33], *GhGA2ox* [34], *GhAGP* [35], *GhGT64* [36], and others.

Based on analyses of preliminary transcriptome data, we found that some genes in the GT4 family are involved in both drought stress responses and fuzz development in cotton. A systematic investigation of this gene family was then carried out across four *Gossypium* genomes, integrating phylogenetic reconstruction, exon–intron organization, conserved motifs, chromosome distribution, gene duplication, collinearity, and Ka/Ks-based selection tests to elucidate their evolutionary affinities. We then performed WGCNA to investigate the interaction networks of this family in drought stress and fuzz development, carried out subcellular localization, and validated expression levels in some extreme materials, thereby preliminarily elucidating the response mechanisms of the GT4 family to drought stress and fuzz development in *G. hirsutum*.

### 4.1. Basic Analysis of GT4 Family Members in G. hirsutum

In this study, we first used the published whole-genome sequences of four cotton species as references and identified a total of 49 GT4 genes. On this basis, we systematically examined the biochemical properties of the proteins encoded by these genes. Specifically, the length of this protein family ranged from 392 to 1153 amino acids, with an average sequence length of approximately 481 aa; the molecular weight ranged from 43.51 kDa to 131.42 kDa, with an average of about 54.08 kDa; and the isoelectric points ranged from 6.06 to 9.59, with an average of 8.23, indicating that they belong to the basic protein category. Although the members display diversity in physicochemical properties, functions, and regulatory mechanisms, they all without exception retain the characteristic GT4 domain, indicating that this domain is highly conserved within the family. To infer phylogenetic relationships among species, we reconstructed a phylogenetic tree, which revealed a notable pattern: GT4 sequences from *G. arboreum* and *G. raimondii* (representatives or close relatives of the diploid progenitor lineages) consistently clustered together with the corresponding sequences from *G. hirsutum* and *G. barbadense* (the tetraploid cultivated species) in the same subgroups. This clustering pattern is consistent with the proposed origin of tetraploid cotton species through hybridization-polyploidization events between the two diploid ancestral lineages [35]. Meanwhile, by analyzing gene structures (intron/exon organization) and protein motif compositions, we found that closely related branches on the phylogenetic tree exhibited high similarity in these features. Chromosomal physical mapping further indicated that, regardless of the diploid or tetraploid background, the relative centromeric positions of GT4 genes on chromosomes exhibited similar patterns.

In addition, we performed prediction of *cis*-regulatory motifs in the promoter regions of the GT4 gene family. The results showed a rich diversity of regulatory elements: hormone-responsive elements included not only methyl jasmonate, salicylic acid, gibberellin, and abscisic acid recognition sequences, but also additional motifs related to ABA signaling cascades; stress-responsive elements covered low-temperature response units, drought-inducible MYB binding sites, defense and wound-stress regulatory boxes, as well as anaerobic response core sequences; light-signaling modules contained both general light-responsive elements and MYB transcription factor-binding light-inducible specific sites. Furthermore, we identified several regulatory motifs controlling endosperm-specific gene expression, meristem activity maintenance, and direct binding to MYBHv1 protein. This comprehensive element annotation provides a key information platform for the regulatory network of this family in complex processes such as development, stress, and metabolism. Regarding gene duplication modes, the statistics showed that in tetraploid cotton species, whole-genome duplication or segmental duplication was the main source of GT4 genes, whereas diploid species exhibited a mixture of whole-genome, segmental, dispersed, and tandem duplications. Finally, we calculated the Ka/Ks ratios for homologous gene pairs at both diploid and tetraploid levels, and all examined values were less than 1. These data suggest that the GT4 gene family has been predominantly subject to purifying selection during species evolution, which may have contributed to limiting the accumulation of deleterious mutations and maintaining functional stability [37,38,39,40]; however, because Ka/Ks values are gene-wide averages, this analysis does not exclude positive selection at specific codons or branches.

### 4.2. Expression Pattern Analysis of GT4 Family Members in G. hirsutum

Based on tissue transcriptomic profiles, developmental time series, and multi-stress response data, the expression patterns of GT4 family members exhibited significant functional divergence. At the organ level, some transcripts (such as *GhGT4_10* and *GhGT4_2*) showed floral organ preference, while others (like *GhGT4_5* and *GhGT4_7*) were enriched in roots and reproductive structures. During ovule and fiber development, most genes showed early active expression followed by downregulation, but some members were re-upregulated during fiber elongation or secondary wall thickening, suggesting their possible involvement in specific stages of fiber morphogenesis. *GhGT4_5* and *GhGT4_9* exhibited negative and positive regulatory tendencies toward fuzz formation, respectively, while the differential dynamics of *GhGT4_15* might participate in the molecular switch for fuzz initiation and differentiation. In abiotic stress responses, *GhGT4_10* and *GhGT4_2* showed strong and temporally specific induction under cold, drought, salt, and heat stresses; in particular, the former peaked under all stress conditions, identifying both as stress-responsive candidate genes that may be relevant to broad-spectrum stress tolerance, pending functional validation. At the biotic stress level, *GhGT4_5* and *GhGT4_13* exhibited sharp and sustained increases in Verticillium wilt-susceptible materials, but only limited and rapidly recovering responses in resistant materials, indicating that this variety-specific expression strongly suggests their involvement in disease susceptibility-related pathways rather than in resistance enhancement. In summary, the GT4 gene family exhibits multidimensional regulatory attributes at the transcriptional level, including spatiotemporal specificity, stress-type dependence, and inter-variety variation. Several members have potential as molecular targets for fiber quality improvement, stress tolerance breeding, or disease resistance breeding, and their biological functions can be further verified by genetic transformation or gene editing approaches.

Through weighted gene co-expression network analysis, we identified multiple co-expression modules highly correlated with specific traits in *G. hirsutum* cultivar TM-1 and in materials related to fuzz development. For drought stress responses, the MEgreen module showed the strongest positive correlation with the 12 h post-treatment time point, and this module contained two GT4 members, *GhGT4_2* and *GhGT4_10*. Functional annotation showed that genes in this module were significantly enriched in chloroplast components, light reaction processes, and the protein translation machinery (ribosomal structure and aminoacyl-tRNA ligase activity), and were also associated with glyoxylate and dicarboxylate metabolism pathways. Subcellular localization experiments showed that *GhGT4_2* localizes to chloroplasts, consistent with its predicted localization; this observation supports a possible chloroplast-associated role but does not establish its function or involvement in a specific pathway. Based on these findings, we hypothesize that *GhGT4_2* and *GhGT4_10* may be associated with the drought adaptation network in cotton through possible involvement in three levels—energy supply, protein synthesis, and metabolic adaptation—potentially via maintenance of photosynthetic organelle stability, translational efficiency, and carbon metabolic remodeling [41,42,43]. In the fuzz development analysis, the MEturquoise module was highly correlated with fuzz phenotype, encompassing three family members: *GhGT4_7*, *GhGT4_9*, and *GhGT4_15*. Further expanding the interaction network with *GhGT4_15* as the core, we identified 503 network-connected genes. Pathway annotation revealed that these interacting genes are mainly involved in nucleocytoplasmic transport, phagosome, and spliceosome processes, among which the nucleocytoplasmic transport pathway has been reported to play a role during cotton fiber elongation. It is known that the nucleocytoplasmic transport pathway is directly involved in cotton fiber development [28]. Therefore, we hypothesize that *GhGT4_15* may be associated with the regulation of RNA export and nucleocytoplasmic shuttling of signaling molecules potentially essential for fiber cell elongation, possibly through cooperation with nucleocytoplasmic transport-related factors, and may play a role in fuzz differentiation [44,45,46].

Collectively, the two independent analyses indicate that different members of the GT4 family were assigned to specific co-expression modules associated with drought response and fuzz development, suggesting that this family may have distinct roles in cotton stress adaptation and fiber quality formation. Among them, the expression and co-expression patterns of *GhGT4_2* and *GhGT4_10* are consistent with possible involvement in abiotic stress responses, potentially through metabolic-energy coordination, whereas *GhGT4_15* may be involved in fiber elongation progression, with its co-expressed genes enriched in the nucleocytoplasmic transport pathway; however, whether it functions through this pathway requires functional validation. These findings provide candidate directions for subsequent transgenic functional validation and molecular breeding utilization of specific members.

## 5. Conclusions

In this study, we systematically identified and characterized 49 GT4 genes across four cotton species (*G. arboreum*, *G. raimondii*, *G. hirsutum*, and *G. barbadense*), with 16 members in *G. hirsutum*. Phylogenetic analysis classified them into four subfamilies and revealed evolutionary conservation between diploid and tetraploid genomes, consistent with polyploid origins. Duplication analysis indicated that lineage-specific expansion of the GT4 family was predominantly driven by whole-genome and segmental duplication. Promoter *cis*-element prediction suggested enrichment of light-, phytohormone-, and stress-responsive regulatory elements. Selection pressure analysis of 100 homologous pairs showed Ka/Ks ratios < 1, indicating predominant purifying selection. Expression profiling and qRT-PCR identified *GhGT4_2* and *GhGT4_10* as drought-responsive candidate genes. WGCNA under drought stress revealed that the MEgreen module was most positively correlated with the 12 h post-treatment time point and was significantly enriched in genes annotated to chloroplast components, photoresponse pathways, ribosomal machinery, and glyoxylate/dicarboxylate metabolism. Subcellular localization showed that *GhGT4_2* localizes to chloroplasts, consistent with its predicted localization and supporting a possible chloroplast-associated role, although its function and pathway involvement remain to be validated. In fuzz development analysis, the MEturquoise module was highly correlated with fuzz phenotype, and a co-expression network centered on *GhGT4_15* identified 503 network-connected genes enriched in nucleocytoplasmic transport, phagosome, and spliceosome pathways. *GhGT4_7* and *GhGT4_15* were associated with fuzz development and represent candidate regulators, including possible negative regulation, pending functional validation. Collectively, these findings provide a systematic catalog of the cotton GT4 family, highlight candidate genes for drought tolerance and fuzz development, and offer a foundation for future functional validation and molecular breeding aimed at improving abiotic stress tolerance and fiber quality in cotton.

## Figures and Tables

**Figure 1:**
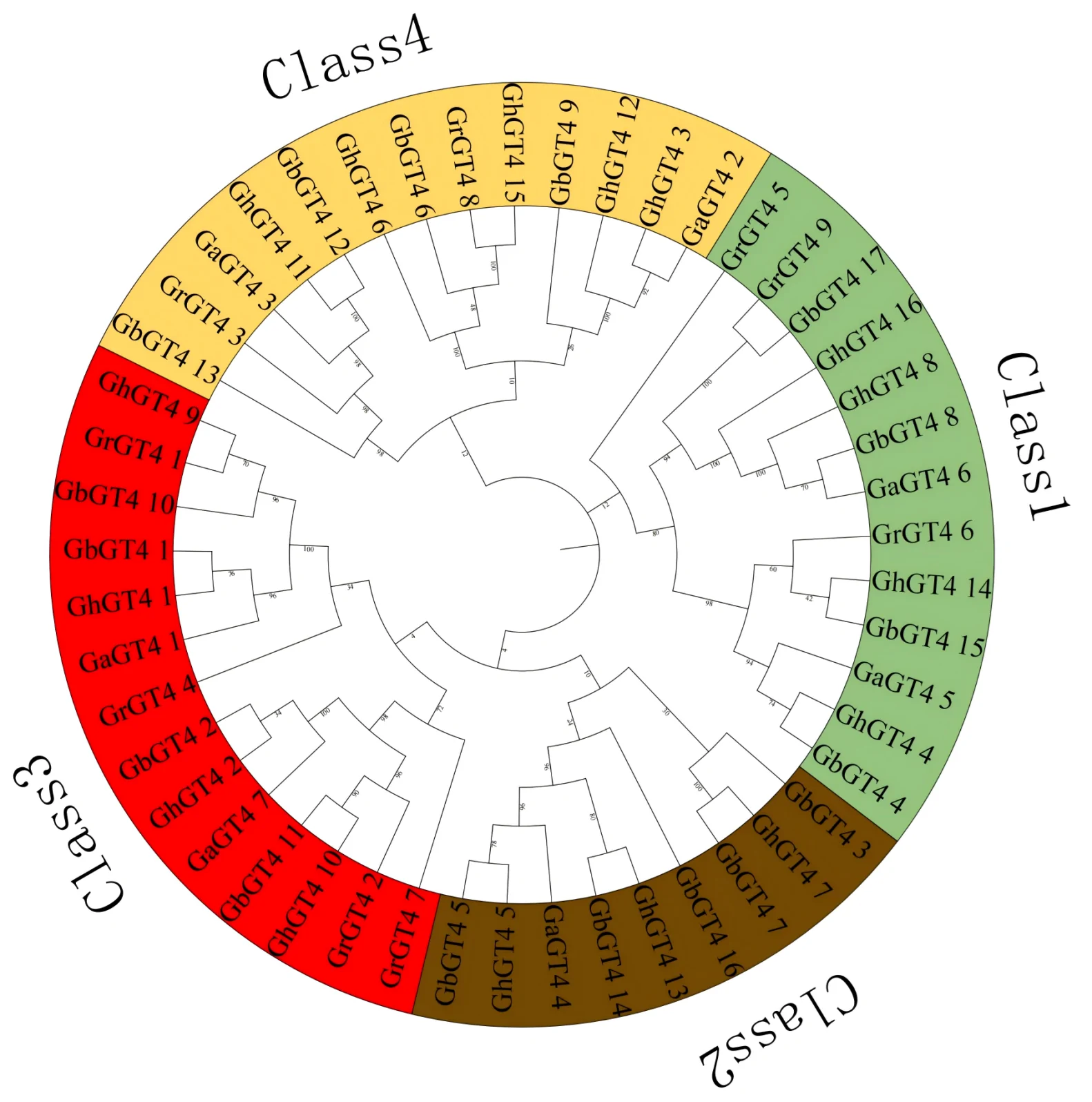
Phylogenetic analysis of GT4 members in four cotton species.

**Figure 2:**
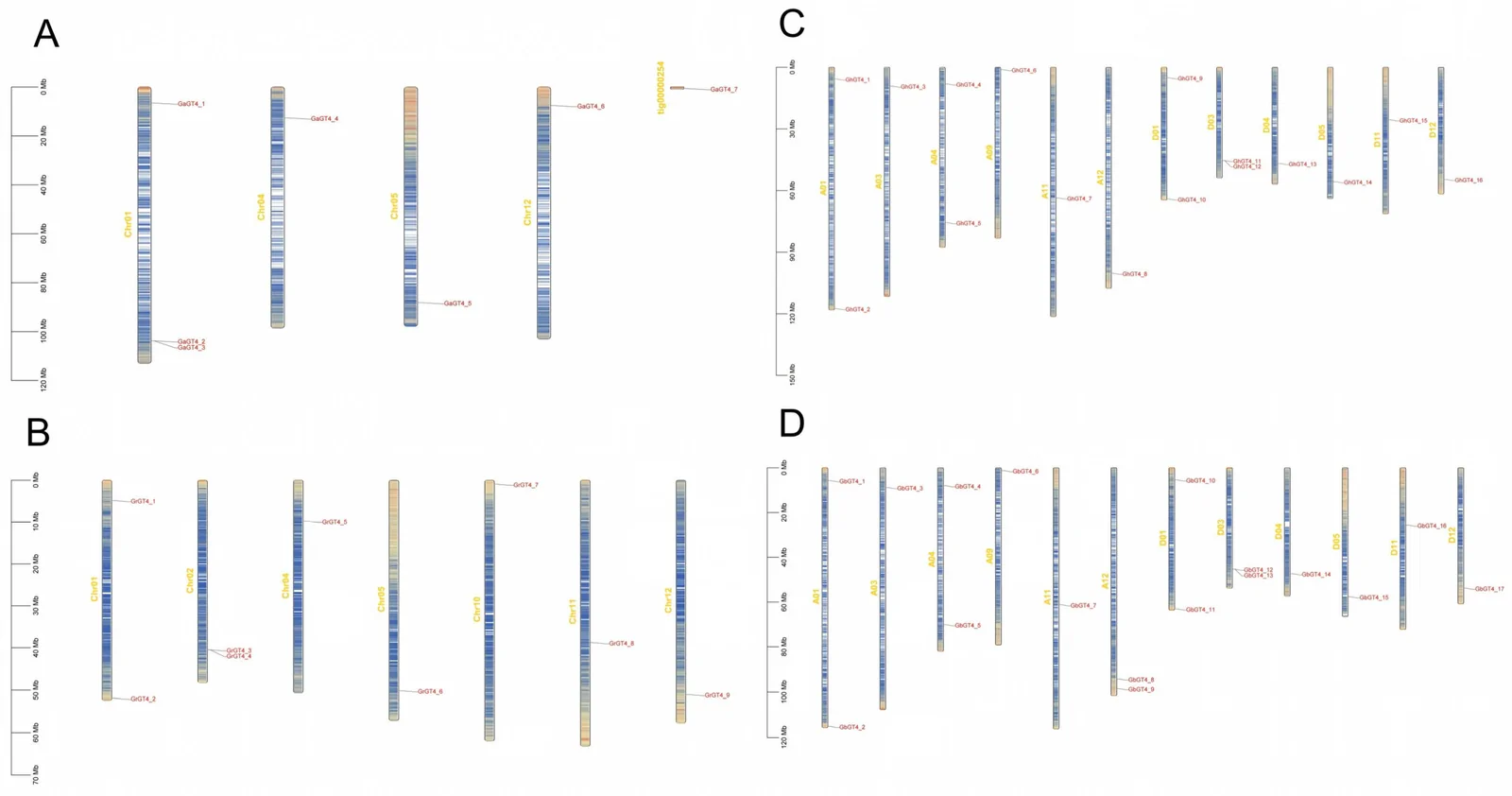
Chromosomal locations of GT4 genes in four cotton species. (**A**) *Gossypium arboreum*. (**B**) *Gossypium raimondii*. (**C**) *Gossypium hirsutum*. (**D**) *Gossypium barbadense*.

**Figure 3:**
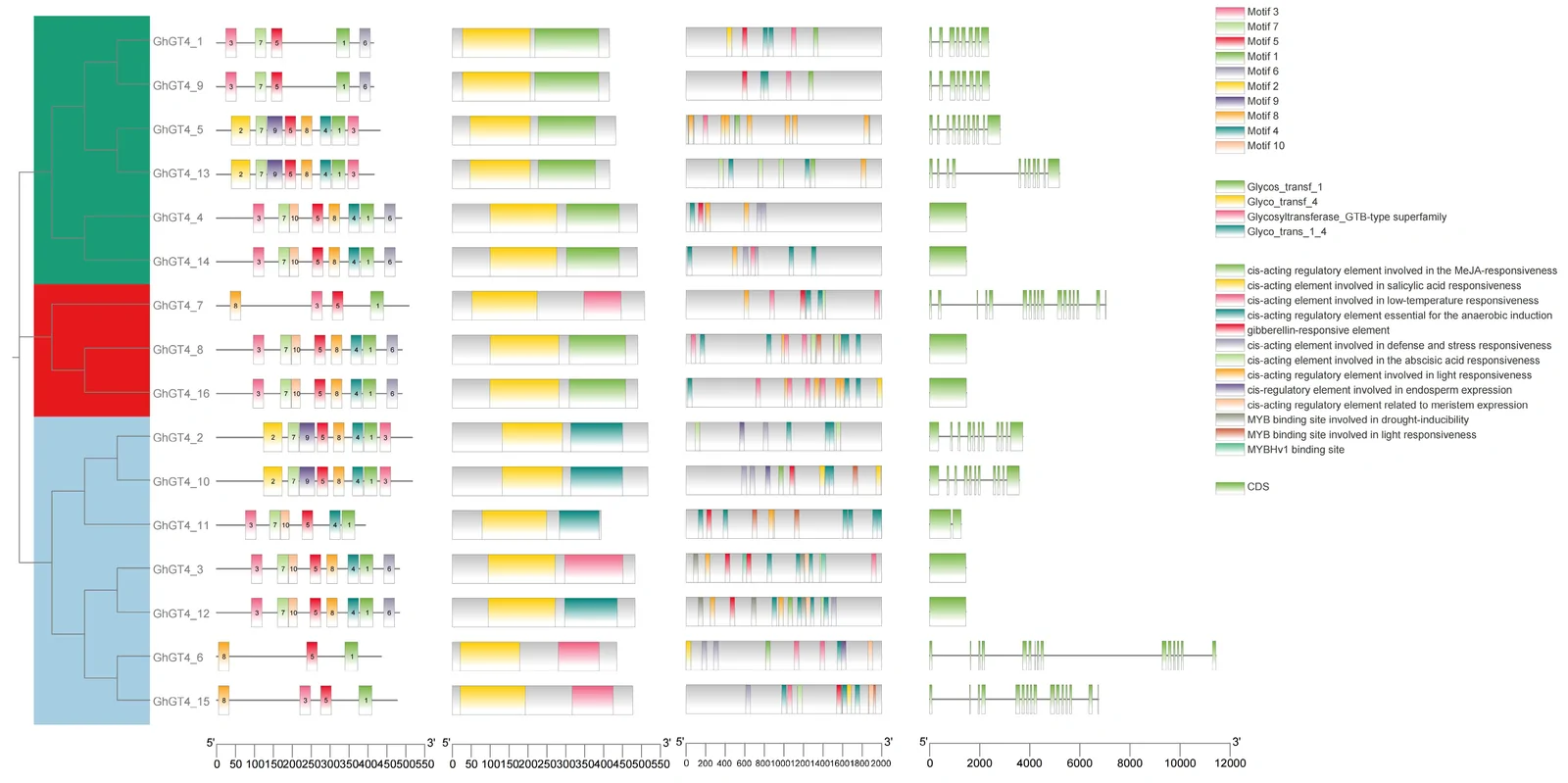
Phylogenetic relationships, architecture, motif composition, and promoter *cis*-elements of the *GhGT4* gene sets.

**Figure 4:**
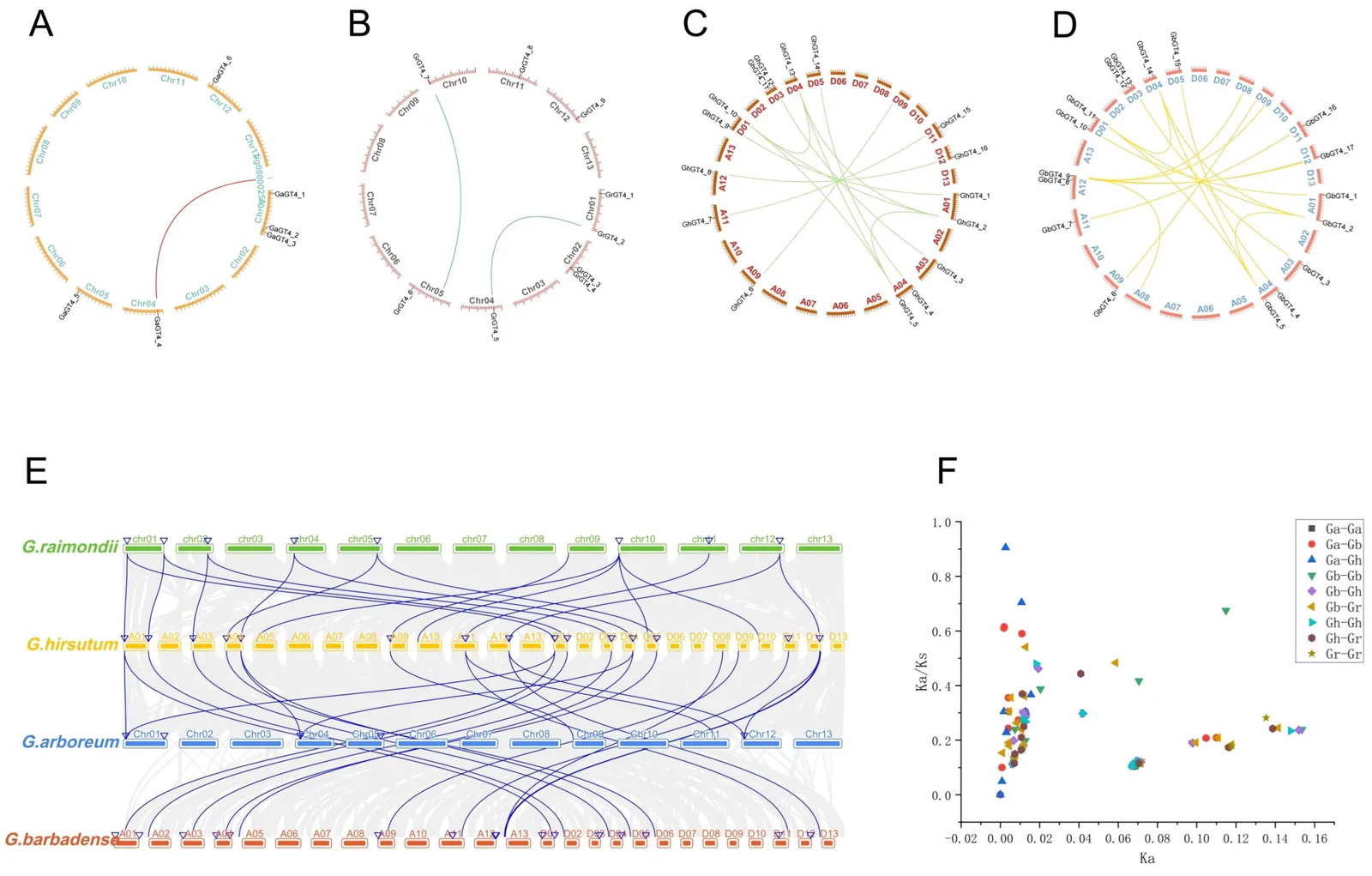
Collinearity, synteny, and selection of GT4 homologs. (**A**–**D**) Collinearity maps for *G. arboreum*, *G. raimondii*, *G. hirsutum*, and *G. barbadense*, respectively. (**E**) Multi-species synteny showing orthologous gene pairs among the four cotton taxa, with chromosomes rendered in species-specific colors. (**F**) Purifying selection inference based on Ka/Ks calculations for GT4 gene pairs.

**Figure 5:**
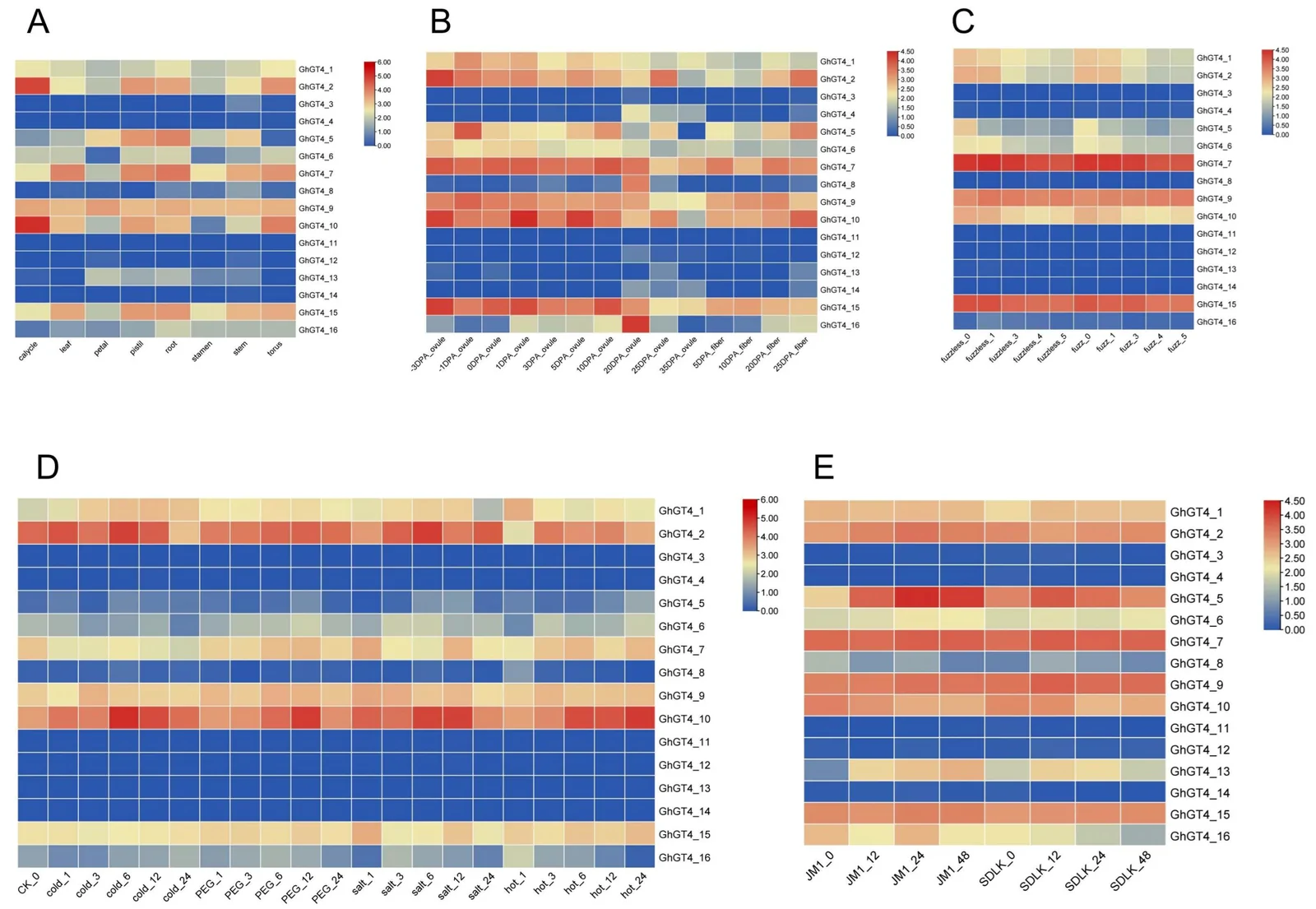
Expression patterns of *GhGT4* genes in diverse tissue types. (**A**) The expression patterns of *GhGT4* genes in diverse organs (Root, Stem, Leaf, Petal, Receptacle, Calycle, Stamen, Pistil). (**B**) Expression trends of *GhGT4* genes in ovules and fibers (−3, −1, 0, 1, 3, 5, 10, 20, 25, and 35 DPA). (**C**) Expression levels of *GhGT4* genes in ovule and fiber in fuzz material (XNZ118) and fuzzless material (XNZ118FLM) at different periods (0,1,3,4,5) DPA. (**D**) Expression footprints of *GhGT4* genes under cold, heat, salt and drought stress conditions (0, 1, 3, 6, 12, 24 h). (**E**) Illustration of *GhGT4s* reaction in *G. hirsutum* when exposed to *Verticillium dahliae* at varying time points.

**Figure 6:**
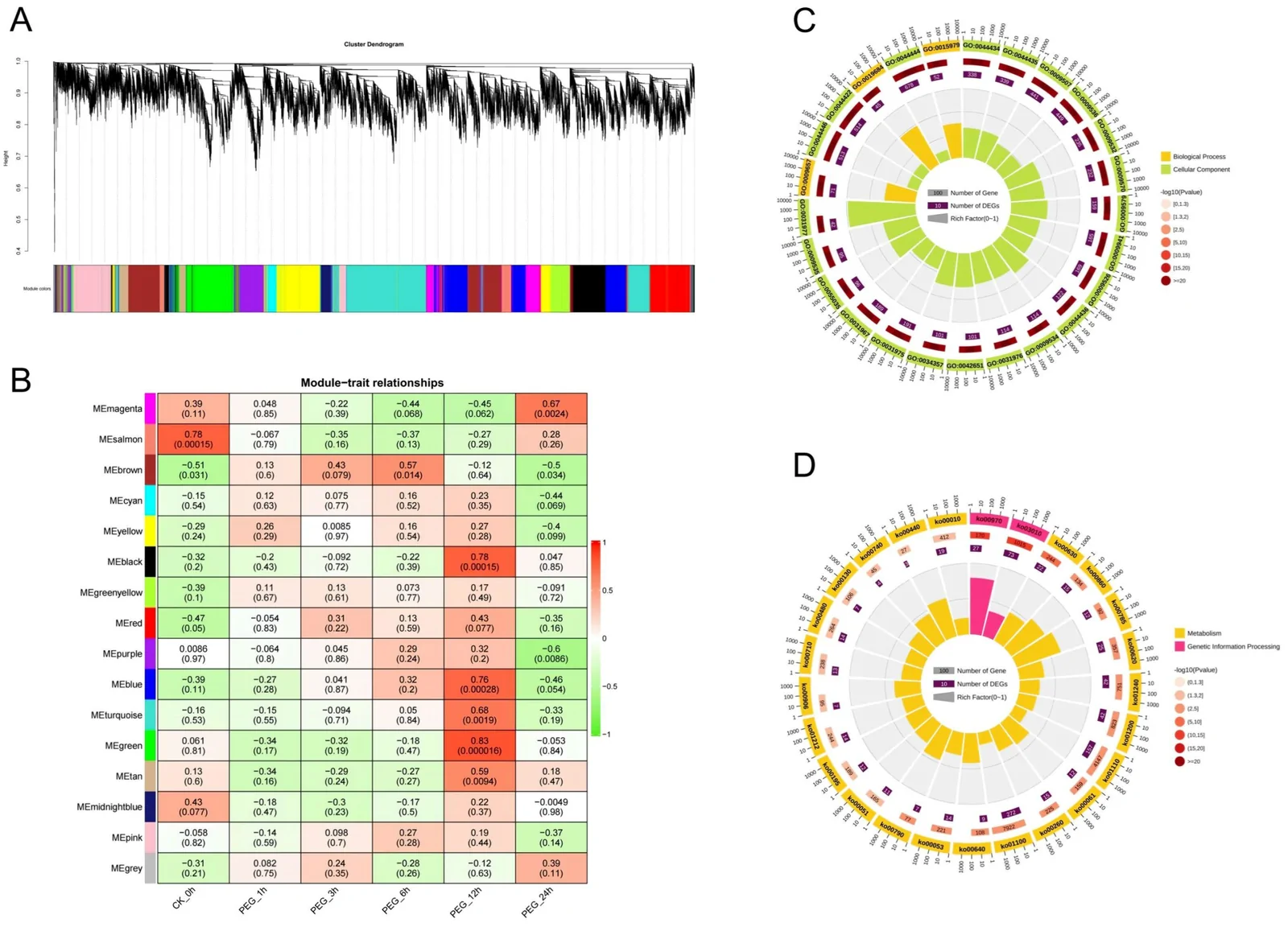
Network topology and functional characterization of drought-associated modules in *G. hirsutum*. (**A**) Clustering of genes by expression similarity. (**B**) Correlations between modules and physiological traits, with r and *p* values indicated. (**C**,**D**) Enrichment of the MEgreen module in GO categories and KEGG pathways, respectively.

**Figure 7:**
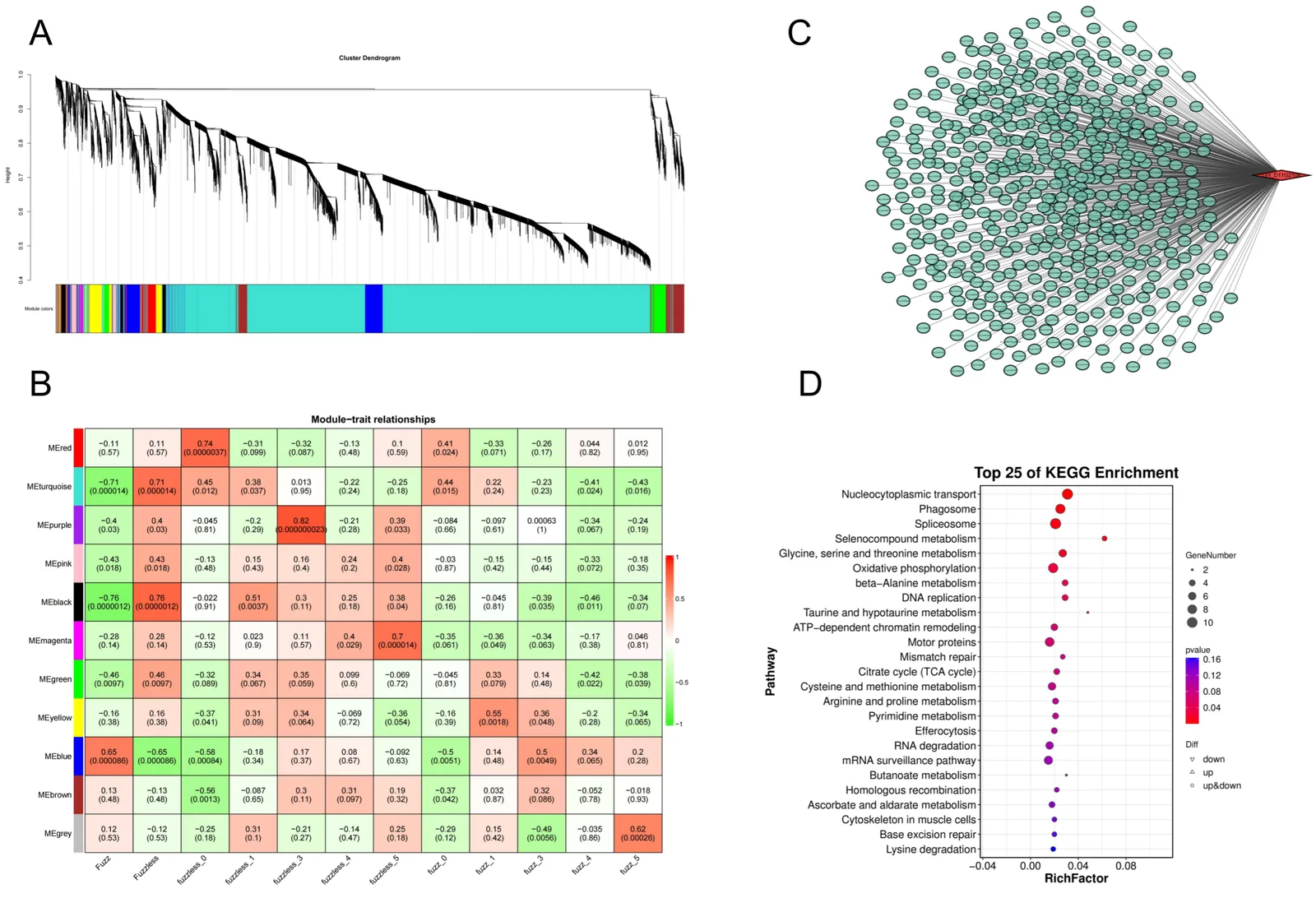
WGCNA analysis in fuzz and fuzzless materials in upland cotton. (**A**) Results of the gene grouping analysis carried out in WGCNA. (**B**) Number of genes within the 11 co-expressed module elements. (**C**) Co-expression network of 503 unique network-connected genes (nodes) co-expressed with *GhGT4_15*. (**D**) KEGG pathway enrichment analysis of network-connected genes co-expressed with *GhGT4_15*.

**Figure 8:**
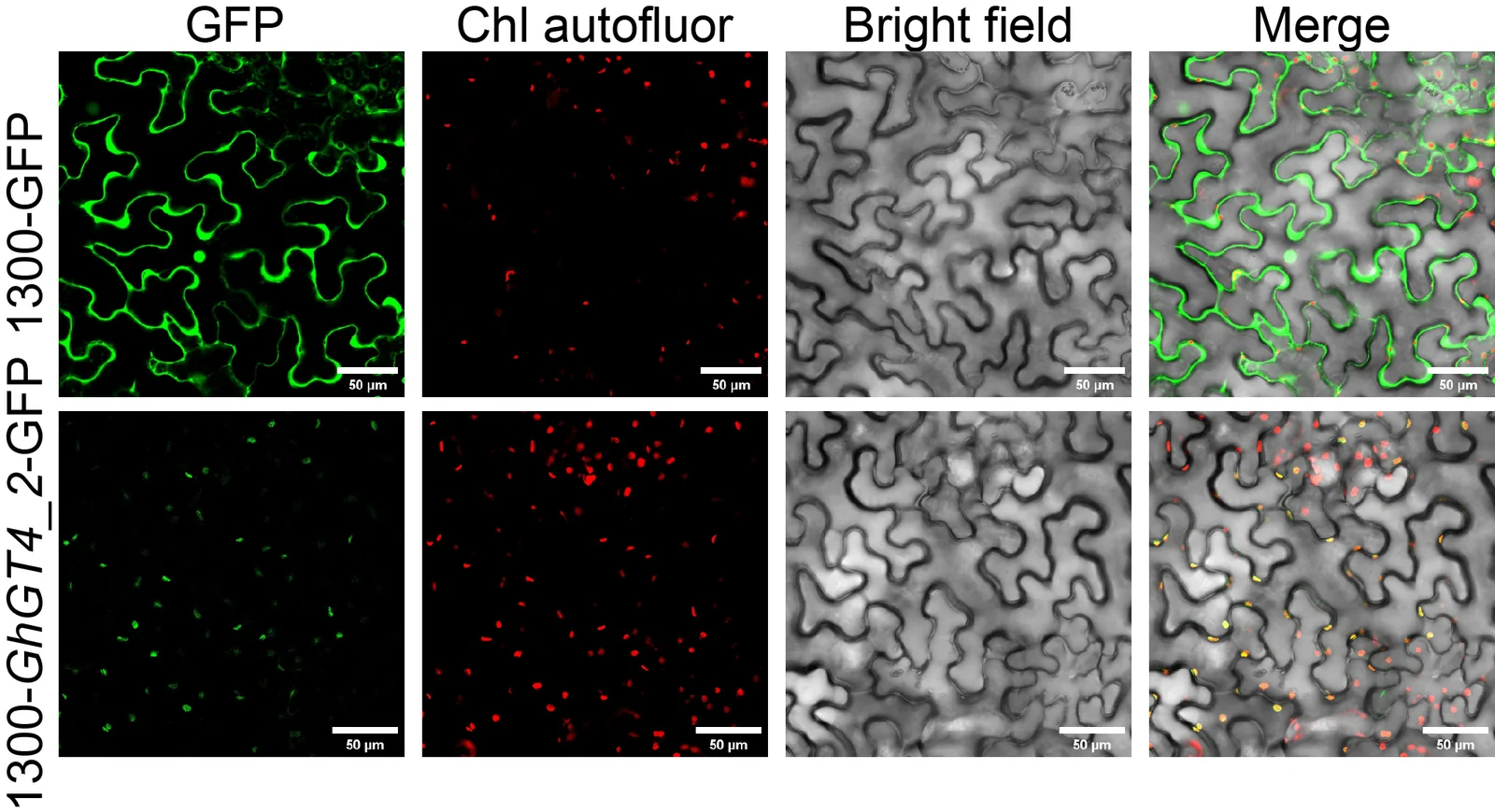
*GhGT4_2* protein subcellular targeting in tobacco leaves. Constructs encoding either free GFP (positive control) or *GhGT4_2*-GFP were agroinfiltrated into epidermal cells. GFP fluorescence was examined 48 h later under a confocal microscope. Red signal indicates chlorophyll autofluorescence (Chl). Bars = 50 µm.

**Figure 9:**
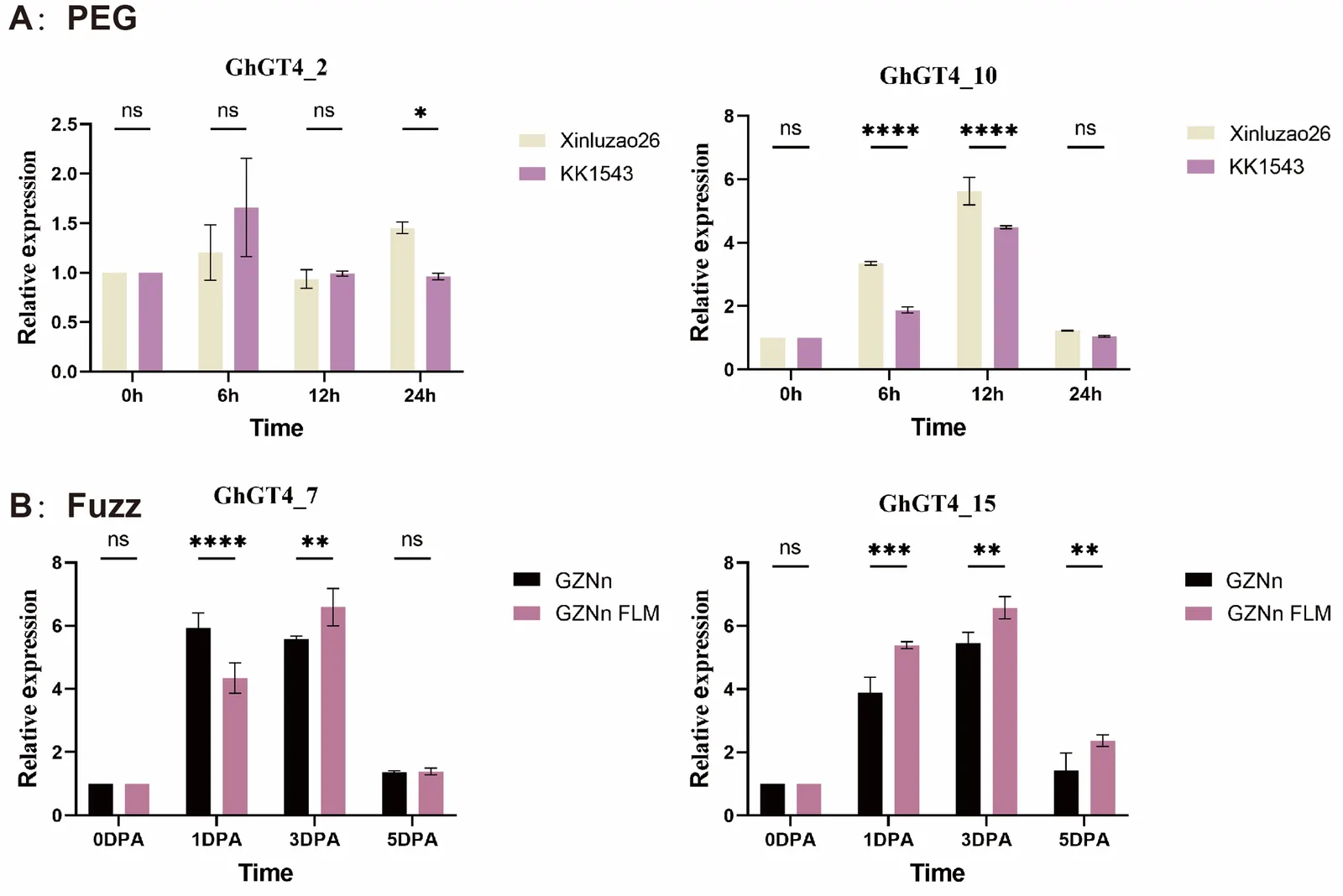
Relative expression analysis of GT4 family members in different cotton materials under different treatments. (**A**) Relative expression levels of *GhGT4_2* and *GhGT4_10* in Xinluzao26 and KK1543 under PEG-simulated drought stress (0, 6, 12, and 24 h). (**B**) Relative expression levels of *GhGT4_7* and *GhGT4_15* in GZNn and GZNn FLM at different stages of fuzz development (0, 1, 3, and 5 DPA). The X-axis represents treatment time (h) or days post anthesis (DPA), and the Y-axis represents relative expression. Error bars represent the mean ± standard deviation (SD) of three biological replicates. Significant differences between different materials at the same time point were analyzed using two-way ANOVA followed by Tukey’s HSD multiple comparison test. Asterisks indicate significance levels (* *p* < 0.05, ** *p* < 0.01, *** *p* < 0.001, **** *p* < 0.0001), and ns indicates no significant difference.

## Data Availability

The RNA-seq data are available at NCBI SRA (https://www.ncbi.nlm.nih.gov/sra, accessed on 8 February 2026) under accession PRJNA248163. Please contact the corresponding author for the remaining data.

## Supplementary Materials

The following supporting information can be downloaded at https://www.mdpi.com/article/10.3390/biology15191709/s1: Figure S1. Correlation heatmap of transcriptome; Table S1. Statistics of RNA-Seq data; Table S2. Primers used for qRT-PCR and Cloning PCR; Table S3. Physicochemical properties of GT4 genes; Table S4. GT4 gene duplication type; Table S5. Ka/Ks Values of all duplicated gene pairs from four *Gossypium* species; Table S6. Interaction network of *GhGT4_15* in the MEturquoise module.
